# Unmet health needs and barriers to health care among people experiencing homelessness in San Francisco’s Mission District: a qualitative study

**DOI:** 10.1186/s12889-022-13499-w

**Published:** 2022-05-30

**Authors:** Anna L. Thorndike, Hailey E. Yetman, Anne N. Thorndike, Mason Jeffrys, Michael Rowe

**Affiliations:** 1 The University of Chicago Pritzker School of Medicine, Chicago, IL USA; 2 Division of Preventive Medicine, Brigham and Women’s Hospital, Boston, MA USA; 3 Division of General Internal Medicine, Massachusetts General Hospital, Boston, MA USA; 4 Harvard Medical School, Boston, MA USA; 5 Mission Neighborhood Health Center, San Francisco, CA USA; 6 Department of Psychiatry, Yale School of Medicine, New Haven, CT USA

**Keywords:** Homelessness, Community-based programs, Access to health care, Health needs assessment, Qualitative research, Patient perspectives

## Abstract

**Background:**

People experiencing homelessness have unique health needs and barriers to medical and behavioral health care (mental health, substance use disorder, and overall well-being) compared to housed people. It remains unclear why many people experiencing homelessness do not access care when community-based homeless health care resources are available at low or no cost. This qualitative study examined perspectives of people experiencing homelessness and staff members at community-based homeless health and service organizations in San Francisco’s Mission District on unmet medical and behavioral health needs and barriers to accessing care.

**Methods:**

We conducted 34 interviews between September and November 2020: 23 with people experiencing homelessness and 11 with staff at community-based homeless health and service organizations in the Mission District. Qualitative interviews were transcribed, coded, and analyzed using the Framework Method on NVivo Qualitative Data Analysis Software.

**Results:**

Both staff and homeless participants reported unmet and common health needs of mental illness, physical injury and disability, food and nutrition insecurity, and substance use disorder. Barriers to care included negative prior health care experiences, competing priorities, and provider turnover. Recommendations for improving services included building more trust with people experiencing homelessness by training clinic staff to treat patients with respect and patience and expanding clinical outreach and health education programs.

**Conclusions:**

People experiencing homelessness face many different health needs and barriers to care, some of which community-based organizations have the ability to address. These findings can help inform future strategies for homeless health care programs to identify and target the specific unmet health needs and barriers to care of people experiencing homelessness in their communities.

People experiencing homelessness (PEH) experience a wide variety of medical and behavioral health needs, many of which remain unmet due to barriers to accessing routine and preventive care. Behavioral health includes mental health, substance use disorder, and overall well-being, as defined in the U.S. by the Centers for Medicare and Medicaid Services [1]. PEH frequently use emergency departments for health care, contributing to overcrowded emergency rooms and significant hospital costs, and experience higher mortality rates than the general population [2–5]. Although there are health care services for PEH in many U.S. cities, these services remain underutilized [6–9]. It is critical that PEH have access to and use community-based, preventive and longitudinal health care services to address their unmet medical and behavioral health needs, supporting their improved quality of life, reduced mortality, and reduced strain on emergency departments.

Most previous qualitative research on unmet health needs and barriers to health care access for PEH has been done outside of the U.S., in countries with universal health insurance [10–13]. Few U.S. studies have explored perceptions of PEH or health care staff about the unmet health needs and barriers to care of PEH [13–15]. Qualitative research provides an accessible platform for homeless individuals to report their personal health care experiences and health care staff to describe their experiences working with this patient population [14, 15]. In a review of 22 studies on homeless persons’ experiences of health care, 8 were qualitative studies conducted in the U.S. [16]. These studies described PEH perceptions of service or outreach workers and overall health care experiences, but there was little or no investigation of PEH perceptions of their own health care needs and barriers to accessing community-based medical and behavioral health care.

Low or no cost health care services from community-based clinics are available to many unhoused individuals in U.S. urban areas, though there are complex, systematic barriers that people must overcome to access health care [9]. The U.S. homeless population increased for four consecutive years prior to the COVID-19 pandemic, estimated at 580,466 people in January 2020 before the pandemic postponed or modified annual citywide counts [17–19]. With a 49% projected rise in chronic homelessness in the U.S. as a result of increased unemployment due to the COVID-19 pandemic, homeless health care services will face increased demand in the coming years [17]. A clearer understanding of the perspectives of PEH and staff at community-based organizations that provide health care to this population is needed to improve and expand existing community-based health care services for homeless Americans.

This study focuses on the health needs and barriers to accessing health care of PEH that persist when community-based homeless health care services are present in an urban area and freely available to unhoused community members. The study took place in the Mission District of San Francisco, CA. The Mission Neighborhood Resource Center, a program of the Mission Neighborhood Health Center, is a community-based medical and behavioral health clinic and day shelter that offers bilingual, free of charge, and drop-in services to individuals experiencing homelessness in the Mission District. The Mission Neighborhood Resource Center is located in the 6th supervisorial district of San Francisco, where 3659 people experiencing sheltered and unsheltered homelessness were recorded in 2019 [20]. The present study conducted interviews with staff at the clinic and at local homeless service organizations, as well as with adults experiencing homelessness in the Mission neighborhood. The objective was to explore the perspectives of staff and PEH regarding the unmet and persistent medical and behavioral health needs of PEH and their access to health services.

## Methods

### Participants and setting

The study took place at the Mission Neighborhood Resource Center and in the Mission District neighborhood in San Francisco, CA. Purposive sampling was used to select two target populations that would provide relevant and distinct perspectives on medical and behavioral health needs of PEH in the Mission District: (1) staff members at the resource center and other local organizations that provide services to PEH and (2) adults 20 years or older experiencing homelessness in the Mission District.

Thirty-four interviews were conducted between September 30, 2020 and November 25, 2020, including 11 interviews with staff members (8 from the resource center, 2 from local homeless service organizations, and 1 staff member at both the resource center and a local service organization) and 23 interviews with adults experiencing homelessness in the Mission District (14 clients at the resource center and 9 non-clients) (Table 1).

**Table 1:** Self-reported demographic information collected from staff and people experiencing homelessness (PEH)

		Staff Members (%)	PEH (%)
		*N* = 11	*N* = 23
Gender Identity	Male	5 (45.5)	15 (65.2)
	Female	6 (54.5)	6 (26.1)
	Transgender Female	0 (0)	1 (4.3)
	Polygender	0 (0)	1 (4.3)
Age	20–29	3 (27.3)	2 (8.7)
	30–39	1 (9.1)	1 (4.3)
	40–49	1 (9.1)	3 (13.0)
	50–59	1 (9.1)	13 (56.5)
	60–69	4 (36.4)	4 (17.4)
	Not Reported	1 (9.1)	0 (0)
Race	Mexican or Central American	4 (36.4)	9 (39.1)
	White	3 (27.3)	3 (13.0)
	Black or African American	2 (18.2)	4 (17.4)
	Mixed	1 (9.1)	2 (8.7)
	Asian	1 (9.1)	1 (4.3)
	Native American or Indigenous	0 (0)	3 (13.0)
	Not Reported	1 (9.1)	1 (4.3)
Ethnicity	Hispanic/Latino	5 (45.4)	9 (39.1)
	Not Hispanic/Latino	6 (54.6)	14 (60.9)

All interviews with staff members were conducted in English. Of the interviews with PEH, 7 (30.4%) were conducted in Spanish and 16 (69.6%) were conducted in English. Interviews took place at the resource center for resource center staff members and by telephone for staff members at three other local organizations that provide services to PEH in the Mission District. Interviews with PEH took place at the resource center and on sidewalks in the neighborhood surrounding the resource center. Data collection concluded when additional information was no longer adding new themes to the topics of interest, indicating thematic saturation [21].

### Measures

Participants were asked to self-report gender, age range by decade, and race and ethnicity. Two semi-structured qualitative interview guides were developed to direct conversations around the research questions for staff interviews and PEH interviews, respectively. Interviews with staff members at organizations serving homeless individuals followed one guide while interviews with PEH followed another guide. Questions differed between guides based on differences in the roles of staff providing services and PEH receiving services.

### Procedures

All recruitment materials, verbal consent forms, and procedures were approved by the Yale University Institutional Review Board (Protocol #2000029059) on September 23, 2020. All study methods were carried out in accordance with relevant guidelines and regulations. Verbal informed consent was obtained from participants as approved by Yale University’s Institutional Review Board. Researchers provided an explanation of the study and its purpose and answered all participant questions prior to asking for consent. Interviews with study participants were conducted by members of the research team without collecting identifying information from the participants. The semi-structured interview guide was designed to identify unmet health needs, barriers to health care, and areas of success or improvement at the community-based homeless health care organizations. Interviews were conducted with staff members in English and with PEH in English or Spanish. Spanish interviews were translated by native Spanish-speaking staff members at the community-based homeless health care organization. Interviews were recorded using handwritten notes or audio recording, depending on the preference of the participant. Interviews which were recorded ranged from 4 to 31 minutes and lasted an average of 14 minutes in length; many PEH had competing priorities that prevented them from being able to participate in longer conversations. Staff participants did not receive compensation for participation. PEH received a food item or a kit of food and hygiene products for participation.

### Data analysis

Interview audio recordings were transcribed using NVivo Transcription and reviewed by a member of the research team. NVivo Qualitative Data Analysis Software Version 12.6.0.959 was used for data coding and analysis. The Framework Method was used for analyzing study data because of its demonstrated usefulness in multi-disciplinary health research [22]. The method involved a seven-stage procedure of transcription, familiarization with data, inductive coding, developing a framework of analysis, applying the framework to the data, charting a framework matrix, and interpreting the data. Two researchers (ALT and HY) independently coded the interview transcripts, or interview notes if the participant did not consent to audio recording, and resolved discrepancies with input from a third researcher (ANT). The framework of analysis was derived from the data during the coding process.

## Results

### Health needs

Staff (S) and PEH described a variety of different unmet and common health needs of adults experiencing homelessness. Four primary themes of health needs were identified across the two groups: (1) untreated mental illness, (2) untreated substance use disorder, (3) food and nutrition insecurity, and (4) physical injury and disability. However, staff participants more frequently discussed clinical diagnoses and treatments while PEH described their personal experiences.

Twenty-nine of the 34 participants (9 staff and 20 PEH) expressed concerns about a lack of available and accessible mental health and psychiatry services for unhoused people. Staff and PEH described a high prevalence of exposure to trauma both prior to becoming homeless and while experiencing homelessness. Staff participants described mental health needs using clinical language for untreated mental illnesses, such as psychotic disorders and mood disorders. As one noted:

*“I would say that there is a lot of those individuals, that present depressive and anxiety symptoms. I would say that they should somehow get some help so that they improve those symptoms.” (S10).*

PEH grounded their mental health concerns in their living conditions and negative experiences of homelessness. One explained the unique and enduring stress of being homeless:

*“It makes me crazy being outside [...] The stress of being homeless makes people crazy. They need a therapist or psychiatrist.” (PEH03).*

Both staff and PEH identified a lack of available mental health providers for PEH. Reasons for this included that many local mental health care providers were not accepting new patients (i.e. had full panels), did not accept public health insurance, or were unable to provide care for individuals without insurance. Some staff participants expressed a desire for increased mental health services and support groups that are culturally and linguistically appropriate for the diverse population of unhoused patients, especially patients who are Spanish-speaking.

Untreated substance use disorder was described as an unmet or common health need by most staff participants and some PEH. Both staff and PEH posited a relationship between mental illness or trauma and substance use disorder among homeless patients. One staff member highlighted the lack of available substance use disorder treatment services at clinics for PEH:

*“I think most clinics, you know, don’t have access to robust substance abuse treatment services.” (S10).*

Another staff member described their understanding of substance use disorder as a mediator between chronic stress and insomnia:

*“I’ve heard many homeless people tell me that’s why they use substances. Because they need sleep. They know they need sleep and they’re just so terrified of being attacked when they’re sleeping on the street that it’s the only way they can zone out enough to actually get a night’s sleep.” (S05).*

While staff generally acknowledged substance use disorder as an unmet health issue, PEH elaborated on specific challenges to changing their behavior after long-term use of substances such as marijuana, methamphetamines, heroin, and cocaine:

*“I do use--I smoke a fair amount of pot, I do edibles, I just prefer smoking it, I’m not really sure why. It’s gross and it’s bad for you but for some reason. I do, I do use methamphetamine.” (PEH21).*

In addition to behavioral health needs, staff and PEH described chronic physical health needs. Many of these needs were similar to the most common health needs of the general, housed U.S. population, such as obesity, diabetes, hypertension, and heart failure. However, staff and PEH frequently reported three physical health needs that were more specific to PEH: (1) food and nutrition insecurity, (2) physical injury, and (3) disability. PEH described having difficulty accessing healthy food, such as a lack of transportation to reach services that provide food. Staff and PEH agreed on the lack of nutritious food available to unhoused people. One homeless participant said:

*“Nutrition is a big thing that I really worry about. Keeping an ice chest and stuff like that to keep everything at the proper temperature is really hard.” (PEH21).*

Physical injury and disability were commonly attributed to street violence or accidents. Staff participants described how disabilities can be health needs and barriers to accessing health care:

*“Many of these people start out with cognitive or intellectual disabilities and then if they have deafness or blindness on top of that, you can just imagine how that complicates their ability to survive, much less access services.” (S05).*

PEH often connected their injuries or disabilities to terrifying stories of fist fights, stabbings, and motor vehicle accidents they had experienced in the community. One said:

*“I got stabbed by some white kids last year. I am very scared walking around.” (PEH19).*

### Barriers to accessing health care

Staff and PEH identified three main themes of barriers to accessing medical and behavioral health care: (1) negative emotions and experiences with health care of PEH, (2) competing priorities of PEH, and (3) health care provider turnover.

Both groups spoke about the emotions of shame, stigma, fear, and a lack of trust or belief in clinical staff or the health care system among PEH. Stigma was linked to receiving behavioral health care, as one staff participant explained:

*“There is that stigma that wanting to get mental health, is somehow, you don’t see it that much because with primary care, you can see a wound or you can see anything that is happening with the health care of the person. But, with mental health, you cannot see.” (S10).*

PEH commonly recounted stories of prior negative experiences with health care, such as poor treatment from providers and staff at community-based health care clinics and at hospitals in San Francisco. One homeless participant expressed frustration about their previous experiences with providers:

*“The way health care providers treat me is horrible. It prevents me from going back. They shame me because I am homeless but I choose to be out here [...] I’m not an idiot. I don’t feel like I can get quality care.” (PEH23).*

Similarly, staff and PEH cited competing priorities as major barriers for PEH when scheduling and attending regular health care appointments. Examples included employment, food, and substance use. Time spent seeking work or income was the most commonly discussed competing priority. Both staff and PEH said that non-medical priorities prevented patients from attending appointments. One PEH commented:

*“Well, sometimes it’s hard to keep up with appointments, if your like main goal is to survive. [ …*] *before going to an appointment, you had to feed yourself and then like you need like transportation to get from one place to another. And if you like--if you don’t get a [...] way to get to your appointment, you’re gonna be late or you won’t go.” (PEH14).*

PEH also noted that lack of transportation was an additional barrier to accessing care.

Both groups noted provider turnover as a barrier to continuing to access health care, highlighting the importance of a primary care provider who is familiar with each patient’s history and life circumstances. One homeless participant spoke of this challenge in detail:

*“Somebody like a doctor or a nurse that gets to know you and you have a rapport with her so she can evaluate if you’re doing worse or if you’ve progressed. See if you have a different one every time, she only goes to the question, goes and [writes it down]. You know, so many-there’s so many homeless people and so many-that we become this, you know, as long as they keep us alive, that’s the thing.” (PEH20).*

Staff participants focused on the implications of provider turnover on the behaviors and health outcomes of homeless patients, such as turnover leading to decreased medication adherence and unhealthy eating habits:

*“So when we had different doctors like every other three months, then patients tend to fall into the cracks and they eventually stop coming or they don’t, I don’t know, they stop taking their medications and stuff like that and also they’re not eating healthy because they don’t have money to do so.” (S07).*

### Recommendations for clinic improvement

Staff and PEH had many shared recommendations for community-based homeless health care services to reach an increased patient population, improve quality of care, or expand available services. The four main themes of recommendations were: (1) building more trust and rapport with patients, (2) expanding clinical outreach, (3) providing health education, and (4) expanding drop-in services to address basic needs.

Both staff and PEH spoke of the need for staff to build more trust with their homeless patients through practicing empathy and respect. One PEH, with the assistance of a staff translator, said:

*“More patience with clients. More respect. Staff needs to give more respect to clients.” (PEH03).*

Staff participants identified lack of patience and respect for homeless patients among some staff members:

*“But yeah, I understand that patients or clients can be difficult, but given where we work, we kind of have to take-we have to kind of to understand them and understand where they’re coming from and maybe they’re having a really, really rough day [...] I think what’s preventing patients from coming back or new patients feel uncomfortable [...] would be reception downstairs and upstairs, the way patients are treated.” (S07).*

Both staff and PEH identified increased clinical outreach as a method for increasing patient numbers and improving community health. PEH reported a need for clinic staff to go out onto the sidewalk and street in the neighborhood and disseminate more information about the services offered at the clinic to other PEH. They also said more frequent outreach would help people who are homeless recognize clinic staff and enhance their willingness to seek services. In contrast, staff participants recommended methods of outreach such as dispensing flyers or flu shots outside the clinic walls:

*“You can make flyers and then, you know, send people out on the street [...] you do a little advertising and then you draw people into the clinic with those, with those things. And then you can use that as an opportunity for other things like screenings.” (S09).*

Staff and PEH talked about the need for more health education (e.g., education on mental health, the dangers of substance use, and understanding medications) for PEH in the community. Both also spoke of the need for health care education tailored to each patient’s cultural, linguistic, health literacy, and cognitive ability status. Staff participants noted that even well-intentioned health education from a provider can be insufficient due to cultural or linguistic limitations:

*“Conditions are not explained in a culturally sensitive manner. Patients don’t understand why they need to take their medications. Providers and staff need to meet the patient where they are at.” (S06).*

Staff and PEH frequently praised the availability of drop-in services for basic needs at the community-based homeless health care clinic, such as showers, laundry, food, hygiene products, and phone charging stations. PEH reported satisfaction with existing drop-in services and a desire for their continuation and expansion. Staff participants agreed and additionally noted that services addressing basic needs provide an important function for the clinic by drawing homeless community members into the building:

*“So, I would say the drop-in center is a good way to attract clients over here, especially homeless clients, because they need some type of place to come to, you know, to well to spend the day usually they are because on the streets. So, it’s a good way to attract the clients to come over here and, little by little, they learn that they can get services from the clinic they can get or case management, too.” (S10).*

## Discussion

This study demonstrates the unique perspectives of community-based health care organization staff and PEH on medical and behavioral health issues of PEH, barriers that make it challenging for PEH to access health care, and needed improvements for community-based homeless health care organizations. Barriers to accessing health care in the community include negative emotions about health care experiences, competing priorities, and provider turnover. Future directions for improving health care for PEH include building more trust between clinic staff and PEH and increasing clinical outreach, health education, and drop-in services for basic needs.

Similar to prior research, participants in this study most frequently reported mental illness, substance use disorder, food and nutrition insecurity, and physical injury and disability health needs. In particular, these findings demonstrate the urgency of prioritizing behavioral health care (e.g., mental health, substance use, and overall well-being) for PEH in the U.S. [23]. A 2019 study in Oakland, CA found that 55.7% of PEH have need for mental health treatment and 72.6% have need for substance use treatment [24]. Further, both staff and PEH in the present study identified and shared their experiences with a wide variety of unmet mental health issues, including PTSD, anxiety, depression, suicidal thoughts, bipolar disorder, psychosis, and substance use disorder. Many of these needs remained unmet due to challenges in accessing psychiatric care, medication, and counseling. Although there is little research on whether developing mental illness precedes becoming homeless, some PEH in the present study attributed their mental illness or substance use to homelessness itself [25]. Homeless health care programs should screen all patients for mental health and substance use disorders and prioritize integrating medical and behavioral health care, hiring more behavioral health providers, and providing mental health education in clinic and through outreach in the community.

PEH participants also described negative past experiences with the health care system and how their competing priorities can lead to care avoidance. These experiences are similar to PEH studied in other countries and settings [11, 26–28]. Negative emotions reported ranged from experiences of shame and stigma about seeking behavioral health care, to fear of medical intervention, to memories of poor treatment from providers or clinic staff. The smaller setting of community-based homeless health care organizations provides an important opportunity to reduce such emotions by ensuring that clinic staff receive training to demonstrate empathy for PEH and create an environment that PEH want to return to. Additionally, PEH in our study stated that they have competing priorities that are rooted in external demands, such as seeking employment or income, finding shelter, acquiring food, or maintaining hygiene. However, these participants also said that the Mission Neighborhood Resource Center successfully helped address their competing priorities by providing drop-in case management and services for basic needs on site. Our findings suggest that drop-in services are an effective strategy for other community-based homeless health care organizations to navigate negative emotions and competing priorities of PEH.

The findings from this study can help inform a framework for community-based homeless health care organizations to implement changes to increase attendance, improve quality of care, and expand health care access for the U.S. homeless population. The study highlights the need for health care providers and organizations to build trust and rapport with PEH in the community. Although trust can be particularly difficult for staff to attain with people who have had multiple negative experiences with health care services [26], our study findings suggested that clinic staff must consistently emphasize respect and patience when working with clients. The themes of increasing clinical outreach and health education in the community to build trust with PEH and to encourage regular follow up care are consistent with prior research about the benefits of health-related street outreach [29]. This study emphasizes that outreach services like street-based care and education by foot or mobile vehicle could reduce barriers for PEH who rarely or do not access health services in buildings, such as people with multimorbidity or physical disability, even when it is located in their local community [30]. Further, our findings suggest that community-based health care organizations providing services for non-medical needs such as showers, laundry, and phone charging contributes to building trust with PEH in the community and is an important opportunity to link PEH with health care services.

Although this study focused on the services that community-based homeless health care clinics provide, mainstream health care services must also become more inclusive and committed to the care of PEH. Maintaining a connection with the mainstream health care system is especially important as PEH transition to housed life [31]. Our findings suggest that mainstream health care services should train staff to demonstrate patience and respect towards PEH and tailor health education resources to the culture, language, and health literacy levels of PEH. In the future, we hope to explore the health care experiences of PEH who are transitioning into housing to inform U.S. policymakers about gaps in their continuity of care.

### Limitations

This study has several limitations. First, it was challenging to recruit staff members at other local organizations that work with individuals experiencing homelessness in the Mission District within the study time frame. As a result, our findings may not adequately reflect the perspectives of staff members at other types of organizations serving homeless individuals. Second, the interviews were not conducted by peer researchers with lived experience of homelessness, who might have been able to provide a more knowledgeable and sensitive approach to qualitative interviews with unhoused individuals [32]. However, clinic staff members with years of experience working with unhoused individuals helped connect the research team with potential participants and explain study goals. Third, the average interview length of 14 minutes was a limitation. Although some interviews lasted up to 30 minutes long, competing priorities for PEH appeared to limit their tolerance for longer interviews. Fourth, because PEH were recruited at the clinic or in the neighborhood surrounding the clinic, we did not reach people who may have had more significant barriers to care and were not seen around the clinic or using clinic services. As a result, the study best represents PEH who spend their time within the vicinity of a community-based homeless health care program.

### Implications for community-based homeless health care organizations

This study provides insight into the health care experiences of PEH and staff members who work at community-based organizations that provide care to PEH. Our findings may help inform policy decisions about how community-based homeless health care programs can address the unmet health needs and barriers to care of the U.S. urban unhoused population. To meet the growing and changing needs of PEH in the U.S., community-based homeless health care organizations should continuously reevaluate the needs and challenges of their patient population and implement changes accordingly.

## Data Availability

The datasets generated and analyzed during the current study are not publicly available due to their containing sensitive, personal information from a small number of participants in a small geographical area but are available from the corresponding author on reasonable request.

## Abbreviations

PEH: People Experiencing Homelessness
U.S.: United States
